# Health worker strikes in Kenya: A symptom of governance inadequacies in a devolved health system

**DOI:** 10.1371/journal.pgph.0006412

**Published:** 2026-10-27

**Authors:** Benjamin Tsofa, Moriasi Nyanchoka, Joshua Munywoki, Fatuma Guleid, Nancy Kagwanja, Lucy Kanya, Francis Wafula, Jacinta Nzinga

**Affiliations:** 1 Health Systems and Research Ethics Department, KEMRI-Wellcome Trust Research Programme, Kilifi, Kenya; 2 Department of Public Health-Pwani University, School of Human and Health Sciences, Kilifi, Kenya; 3 Health Economics Research Unit, KEMRI-Wellcome Trust Research Programme, Nairobi, Kenya; 4 Department of Health Policy, London School of Economics and Political Science, London, United Kingdom; 5 Institute of Healthcare Management, Strathmore University Business School, Nairobi, Kenya; 6 Liverpool School of Tropical Medicine, Liverpool, United Kingdom; The University of British Columbia, CANADA

## Abstract

Recurrent health worker strikes in Kenya since the 2013 devolution reforms reflect more than episodic labour unrest; they signal broader governance challenges that may be particularly pronounced in decentralised and resource-constrained health systems. Despite constitutional protections for labour rights and decentralised service delivery, Kenya’s health sector has experienced persistent industrial disputes, exposing gaps in legal frameworks, institutional coordination, and dispute resolution systems. We conducted a qualitative policy analysis of health worker strikes in Kenya between 2010 and 2025. Data were drawn from legal statutes, policy documents, government reports, peer-reviewed literature, and grey sources. Guided by Walt and Gilson’s policy triangle, complemented by complex adaptive systems and health governance frameworks, we analysed how institutional arrangements, stakeholder dynamics, and policy processes shape labour relations in a devolved health system. Several interrelated drivers of recurrent strikes were identified, including tensions between the constitutional rights to strike and to health, fragmented governance and weak intergovernmental coordination under devolution, persistent workforce challenges and failures to implement Collective Bargaining Agreements (CBAs) and Return-to-Work Formulae (RTWFs), ineffective dispute-resolution mechanisms, and the absence of enforceable minimum service arrangements during industrial action. Through their interaction, these factors generate recurring cycles of non-compliance, labour unrest, and institutional mistrust. Recurrent disputes over CBA and RTWF implementation suggest that the central issue lies not in the absence of legal protections or negotiated settlements, but in translating commitments into sustained action. Addressing recurrent strikes requires systemic reform beyond short-term dispute settlement to address underlying governance, accountability, and implementation challenges. Priorities include strengthening independent dispute resolution mechanisms, clarifying responsibilities across levels of government, improving accountability for negotiated commitments, harmonising human resource management systems, and institutionalising minimum service protections. Kenya’s experience offers important lessons for decentralised health systems on aligning authority, accountability, and implementation capacity to strengthen resilience and advance universal health coverage.

## Introduction

Health workers’ (HW) strikes, or industrial actions, have become a central concern in health systems research, not only because they disrupt service delivery, but also because they expose broader governance challenges. Across both high-income countries (HICs) and low- and middle-income countries (LMICs), particularly in Africa, industrial action has emerged as a recurring challenge that undermines equity in service delivery, continuity of care, and progress toward universal health coverage (UHC), revealing how institutions respond under strain [1–6]. Strikes are therefore not isolated labour disputes but signals of systemic dysfunction, making them an important lens for analysing health system resilience and governance.

In Kenya and many other LMICs, HW strikes often reflect broader governance and health system challenges rather than isolated labour disputes [1,7–11]. Grievances span across multiple domains of the health system, including governance, financing, human resources, health products and technologies, and service delivery [12–14]. Common triggers include poor working conditions, delayed or inadequate remuneration, and weak enforcement of Collective Bargaining Agreements (CBAs) [1,4,6,15–17]. These challenges intensify during public health emergencies, such as the COVID-19 pandemic, when health workers face heightened risks and resource constraints rise [18,19].

The consequences of HW strikes are multi-layered. For patients, strikes reduce access to care, drive out-of-pocket spending when private care substitutes, and worsen health outcomes, particularly among vulnerable groups [12–14,20]. For facilities, they cause operational disruption, strain staff relations, and erode public trust [11,12,21]. System-wide, strikes widen health inequities, stalls progress toward UHC, and interrupt medical education and training [3,16,21,22]. Severity varies with strike duration, scope, cadres involved, and the availability of alternative or minimum services care [6,12,14], factors that are shaped by governance design and the strength (or weak enforcement) of continuity safeguards. These strikes also surface ethical and legal tensions [4,6,18,20,23,24]. The right to strike is protected under international law, including International Labour Organization (ILO) Convention 151, yet this right must be balanced against the duty to safeguard patient welfare [4,16,17,24,25]. Ethical debates often centre on whether industrial action in healthcare can be justified when long-term improvements in working conditions and service delivery are weighed against short-term harm to patients [18,24]. Resolving this tension requires more than moral argumentation; it demands coherent governance and policy reforms that align labour rights with institutional resilience through clear legal frameworks and effective dispute resolution. Professional associations, trade unions, and regulatory councils shape negotiation processes with government authorities and strike outcomes [1,5,16]. Some countries, taking action in line with international labour standards, have adopted minimum service agreements to preserve critical services during industrial action, offering institutional models that reconcile labour rights with continuity of care [5].

Kenya offers a critical lens on these dynamics. Since 2010, recurrent national and county-level HW strikes have exposed fragmented arrangements in health workforce governance, capacity gaps in counties, and inconsistent CBA implementation [10,26–29]. Following the devolution of health services in 2013, the responsibility of employment, management, and negotiation shifted across multiple institutions. County public service boards employ and manage HWs within their respective counties; County Departments of Health (CDoH) oversee service delivery operations; the Council of Governors (CoG) coordinates inter-county positions; and national ministries, particularly the Ministries of Health and Labour, together with the Salaries and Remuneration Commission (SRC), influence standards, remuneration, and policy. Ambiguity over who negotiates, implements, and enforces CBAs between HW unions, county public service boards, national ministries, and SRC has contributed to weak enforcement and uneven dispute resolution. Political interference and the absence of a robust, institutionalised conciliation mechanism further entrench cycles of unrest, with county variation in capacity and commitment shaping outcomes [30]. These trends have become a growing concern for policymakers, researchers, and health-sector leaders and stakeholders.

Guided by a policy analysis approach, this paper examines Kenya’s health workforce governance, legal and policy environment, and the drivers and context of public sector HW strikes. We examine why labour disputes persist despite existing legal, governance, and dispute-resolution mechanisms and identify reforms to strengthen dispute prevention and resolution. We ask: *Why do recurrent health worker strikes persist in Kenya despite repeated policy reforms, dispute-resolution mechanisms, and negotiated agreements, and how do governance, legal, and institutional arrangements contribute to this persistence?* We address this question by synthesising evidence on strike drivers, analysing governance and legal arrangements, and identifying mechanisms that sustain cycles of agreement, non-implementation, and industrial action.

This study advances the literature on health workforce governance, with relevance to LMICs and decentralised health system contexts, in three ways. First, it provides a longitudinal policy analysis of health worker strikes in Kenya from 2010 to 2025, shifting focus from episodic disputes to the systemic conditions that sustain recurrent labour unrest. Second, drawing on policy analysis, complex adaptive systems, and health governance perspectives, it shows how fragmented mandates, weak accountability, and unresolved tensions between labour rights and service continuity undermine health system resilience. Third, our study identifies actionable reform levers in dispute resolution, intergovernmental coordination, and minimum service protections to strengthen accountability, labour relations, and health system resilience in decentralised settings.

## Methods

### Ethics statement

Ethical approval was obtained from the KEMRI Scientific and Ethics Review Unit (Ref: KEMRI/SERU/CGMR-C/4354), with additional permissions secured for access to various government data sets prior to study commencement. The study used only publicly available secondary data and published literature; no human participants were involved, and no further approval was required. All sources were cited, and analyses followed principles of transparency and reproducibility.

### Study design

We conducted a qualitative policy analysis to examine the governance, legal, and institutional drivers of recurrent HW strikes in Kenya. We grounded our analysis in Walt and Gilson’s policy triangle framework [31], which explores health policy through the interaction of context, content, process, and actors. To deepen our inquiry, we incorporated principles from Complex Adaptive Systems (CAS) theory [32] and the health governance framework [33], which enabled us to explore how decentralisation, institutional arrangements, and stakeholder dynamics shape labour relations in the health sector.

### Analytical framework integration

We applied a multi-theoretical framework to guide our analysis. We used the policy triangle to structure descriptive coding of policy elements, focusing on actors, context, content, and process. We drew on CAS theory to interpret system-level dynamics, including feedback loops, emergent behaviours, and adaptive responses to labour unrest. We also used a health governance framework to assess institutional accountability, decentralization effects, and negotiation mechanisms within Kenya’s devolved health system. This layered approach allowed us to examine both the structural and dynamic dimensions of health workforce governance.

### Data sources, scope, and selection

We conducted a structured review of secondary data sources published between 2010 and 2025, a period capturing the post-constitutional and post-devolution transformation of Kenya’s health system. This timeframe includes major reforms introduced by the 2010 Constitution, particularly the devolution of health services to county governments, which redefined institutional roles, labour relations, and governance structures.

Our data sources included national legal statutes, policy guidelines, government reports, peer-reviewed literature, and grey literature from professional associations, civil society organisations, development partners, media outlets, and union communications. Documents were identified through structured searches of academic databases (including PubMed and Google Scholar), official government and institutional websites, legal repositories, and targeted searches of grey literature. Search terms combined keywords related to health workers, strikes, labour relations, governance, devolution, and dispute resolution.

Documents were purposively selected based on their relevance to health workforce governance, labour relations, strike dynamics, institutional mandates, legal frameworks, dispute resolution mechanisms, and stakeholder perspectives. Approximately 75 documents met these criteria and were included in the analysis (Table 1). Together, these sources provided complementary evidence on formal governance arrangements and the experiences, positions, and actions of key stakeholders involved in health workforce disputes. Comparative literature from other decentralised LMIC settings was also reviewed to provide contextual insights and support interpretation of the findings.

**Table 1:** Summary of data sources and their analytical contributions.

Document Category	Number Reviewed	Representative Documents	Contribution to Analysis	Level of Governance
National Legal Frameworks	8	• Constitution of Kenya, 2010 • The Employment Act, 2007 • The Occupational Safety and Health Act, 2007 • County Governments Act • Salaries and Remuneration Commission Act • Labour Relations Act, 2007 • The Labour Institutions Act, 2007 • Employment and Labour Relations Court Act	Enabled legal mapping of jurisdictional authority, enforcement gaps, and statutory protections	National
County-relevant Legal and Policy Frameworks	1	• County Public Service Human Resource Manual	Clarified county mandates and HRM responsibilities under devolution	County
Policy Guidelines and Manual	2	• Devolved HRM Policy Guidelines on Human Resources for Health [34] • Public Service Commission Guidelines for mediation, conciliation and mediation	Clarified governance mandates and procedural expectations for dispute resolution and workforce management	Cross-cutting
Government and Agency Reports	12	• Kenya Health Workforce Report: The Status of Healthcare Professionals in Kenya, 2015 [35] • Technical Paper on Management of the National Wage Bill through Effective Labour Relations in the Health Sector • Health Labour Market Analysis for Kenya (2023) • Labour Rights Legal Framework in Kenya	Provided empirical data on workforce composition, fiscal pressures, and policy responses	National and County
Grey Literature and Institutional reports	13	• Reports from policy and research institutes, and development partners • Publications from professional associations and civil society • Dissertation repositories and media archives	Added contextual depth and practitioner insights; surfaced undocumented grievances and informal practices	Cross-cutting
Peer-Reviewed Studies	26	• Studies on health devolution, governance, health labour disputes and HRH in Kenya	Strengthened theoretical framing and comparative analysis; validated findings with scholarly perspectives	Cross-cutting
Union communications and Media reports	13	• Press statements, strike notices, media coverage, public communications	Documentation of strike events, stakeholder positions, implementation challenges	Cross-cutting

Table 1 provides a breakdown of the categories, scope, and analytical contributions of documents reviewed. This mapping reflects the breadth of Kenya’s legal, policy, institutional, and stakeholder landscape and illustrates how decentralisation and fragmented authority shape health workforce governance.

### Data extraction and analysis

Data extraction focused on legal provisions, institutional mandates, governance arrangements, stakeholder roles, dispute resolution processes, and documented strike events. Data from each document was entered into a structured extraction matrix to facilitate comparison across source types and governance levels.

Analysis was primarily deductive and guided by Walt and Gilson’s policy triangle framework [31], which provided an organising structure for examining policy context, content, actors, and processes associated with health workforce governance and strike management in Kenya. Concepts from complex adaptive systems (CAS) theory [32] were subsequently applied as a complementary interpretive lens to explore system interactions, interdependencies, adaptation, feedback processes, and coordination challenges across national and county levels.

Initial coding was undertaken by MN and JM using a coding framework derived from the study objectives and analytical frameworks. Coded data and emerging thematic categories were discussed among co-authors and reviewed by BT and JN with expertise in health policy and systems research and human resources for health (HRH). Emerging codes not adequately captured by the initial framework were discussed and incorporated where they enhanced explanatory depth and relevance.

Within the framework domains, coded data were compared iteratively across documents to identify recurring patterns, areas of convergence and divergence, and relationships between governance structures and strike dynamics. Differences in interpretation were resolved through discussion and consensus, with reference to the original documentary sources. Attention was given to inconsistencies between formal governance arrangements and their implementation in practice.

Findings were triangulated across legal documents, policy reports, government publications, union communications, media reports, and empirical studies. An audit trail documenting refinements to the coding framework and thematic interpretations was maintained throughout the analysis. Comparative literature from other decentralised LMICs were used to contextualise Kenya’s experience and identify potentially transferable policy lessons.

### Reflexivity and validity considerations

The research team comprises health systems and policy researchers with prior experience in Kenya’s health sector. To minimise interpretive bias, we relied on transparent documentary sources and a structured analytical approach guided by explicit theoretical frameworks, documented coding procedures, and reflective discussions throughout the study. Interpretations were critically reviewed with the research team to challenge assumptions and enhance analytical credibility. While the study relied on secondary data, the breadth of documentary evidence and structured analytical approach strengthened the credibility and policy relevance of the findings.

## Results

### Governance and actor fragmentation under devolution

Our study identified patterns of fragmented authority and coordination challenges within Kenya’s devolved health governance arrangements, which may contribute to persistent difficulties in health workforce management and dispute resolution.

From our analysis, we find that Kenya’s post-2010 devolved governance structure has fundamentally reshaped health workforce management, but without sufficiently clarifying roles, accountability, or coordination mechanisms across levels of government. While county governments assumed responsibility for health service delivery and workforce management in 2013, national institutions retained influence over policy direction, remuneration standards, and labour regulation [8,36–41]. The resulting multi-actor landscape summarised in Table 2 is characterised by overlapping mandates and potentially blurred lines of authority that may complicate routine workforce management and labour negotiations.

**Table 2:** Key Actors and Roles in Health Worker Management in Kenya.

Functional Domain	Stakeholder	Roles and Responsibilities	Governance level
Policy Oversight	Ministry of Health (MoH)	Formulates national health policies, including those related to health workforce governance	National
	Public Service Commission (PSC)	Provides oversight and policy guidance on public service management across both levels of government	National
	County Department of Health (CDoH)	Manages promotions, study leave approvals, major disciplinary actions, and intra-county transfers	County
	County Assembly	Provides legislative oversight and budget approval for county health workforce operations	County
Human Resource Management	Council of Governors Secretariat	Coordinates inter-county transfers and harmonisation of public service practices, including health workforce mobility	National (inter-county)
	County Public Service Board (CPSB)	Responsible for recruitment, deployment, and dismissal of county-employed health workers	County
	Health Facility Management	Oversees minor disciplinary actions, sick leave approvals, and routine HR functions such as annual leave processing	County
Regulation and Remuneration	Salaries and Remuneration Commission (SRC)	Determines and reviews wages and allowances for all public servants, including health workers	National
	Health Workforce Regulatory Bodies	Regulate professional standards and licensure for specific health cadres	National
Representation and Advocacy	Health Worker Unions	Represent members in collective bargaining; advocate for improved conditions; organise industrial action	National and County
	Health Professional Associations	Promote professional development, standards, and welfare of their respective members	National and County
	Development Partners	Support employment and secondment of selected health workers; provide technical assistance	Cross-cutting

Sources: (MoH, 2015a, 2015b; Tsofa *et al.*, 2017; MoH, 2019; Munywoki *et al.*, 2020; Koon, 2021).

Note: Some stakeholders operate across both national and county levels.

Our findings suggest that the rapid implementation of devolution may have exceeded the preparedness of some institutions, particularly at the county level, contributing to early-day challenges in salary administration [8,29,42], reporting structures [8], [26], [29], and managerial oversight [8,29,41,42]. These coordination failures were not transient [8,29,42]. Over time, variations in county capacity, fiscal constraints, and intergovernmental coordination challenges appear to have been associated with disparities in human resource management practices, contributing to recurrent labour disputes and eroding workforce morale [7–10]. The finding suggests that national and county institutions do not always operate in a coordinated manner. This may limit opportunities for effective dispute prevention and resolution [40,43,44].

### Labour rights, unionisation, and dispute dynamics

Our analysis suggests that expansion of labour rights and unionisation following the Constitution of Kenya 2010 created greater opportunities for collective organisation and collective bargaining within the health sector. At the same time, these developments occurred alongside evolving governance arrangements and dispute-management mechanisms that may not always have been adequately equipped to manage increasingly complex labour relations within a devolved health system [27,36,44]. Our findings suggest that labour disputes increasingly became a mechanism through which unresolved governance and employment challenges were expressed, although other labour relations, political, and collective bargaining dynamics may also have influenced strike activity.

Table 3 reveals a clear evolution in HW strikes between 2010 and 2025. Before devolution, industrial action was largely concentrated among individual professional cadres and centred on remuneration, working conditions, and union recognition. Following devolution, however, labour disputes increasingly reflected HW governance challenges associated with fragmented employer authority, unclear accountability arrangements, and difficulties implementing negotiated agreements across national and county governments [60–65].

**Table 3:** Summary of nationwide health worker strikes in Kenya.

Cadres Involved	Start Date	End Date	Duration (days)	Grievance Category	Primary Grievances
**Pre-Devolution Period (2011 – 2022)**					
Doctors (KMPDU)	Dec 5, 2011	Dec 13, 2011	9	Union recognition, Remuneration	Poor working conditions, low salaries, and demand for union recognition [7]
Nurses (KNUNM)	1 Mar 2012	15 Mar 2012	15	Remuneration, Working conditions	Salary increase and improved working conditions [7,45,46]
Doctors (KMPDU)	13 Sep 2012	4 Oct 2012	22	CBA Implementation, Infrastructure	Failure to honour the 2011 RTWF, poor hospital infrastructure and resources, call for health sector funding and workforce expansion [7,47]
**Early Devolution Period (2013 – 2017)**					
Nurses (KNUNM)	3 Dec 2012	11 Feb 2013	70	Union Recognition, Remuneration, Equipment	Demand for union registration and recognition (KNUNM), salary and allowances improvements, modern equipment [7,48]
Doctors and Nurses (KMPDU & KNUNM)	Dec 10, 2013	Dec 23, 2013	14	CBA implementation, Devolved governance crisis	Unfulfilled 2013 CBA, low wages, workforce shortages, governance crisis [7]
Doctors (KMPDU)	Dec 5, 2016	Mar 15, 2017	100	CBA Implementation, Working conditions	Non-implementation of the 2013 CBA, poor remuneration, inadequate working conditions [10,29]
Nurses (KNUNM)	Dec 5, 2016	Dec 14, 2016	10	CBA Implementation	Pay and allowance disparities, stalled CBA, poor governance [10]
Nurses (KNUNM)	Jun 5, 2017	Nov 2, 2017	150	CBA Implementation	Failure by national and county governments to sign a CBA [10,29,49]
Clinical Officers (KUCO)	Sep 15, 2017	Oct 5, 2017	20	Remuneration, Career progression	Poor pay, lack of promotions, delayed salaries [29,49]
**COVID-Era Strikes (2020–2021)**					
Clinical Officers & Nurses (KUCO & KNUNM)	Dec 7, 2020	Feb 24, 2021	70	COVID-19 Response, Risk allowances	Lack of PPE, inadequate health cover, absence of COVID-19 risk allowances [27]
Doctors (KMPDU)	Dec 21, 2020	Dec 24, 2020	3	COVID-19 Response, Medical doctors intern management	COVID-19 response gaps, delayed medical intern postings, lack of risk allowance [27,50]
**Post-COVID and UHC-Linked Strikes (2024–2025)**					
Doctors (KMPDU)	Mar 13, 2024	May 8, 2024	56	CBA Implementation, Medical doctors’ interns’ stipends	Incomplete execution of 2017 CBA, medical intern doctors’ stipends disputes, and unsettled salary arrears [51]
Medical laboratory officers (KNUMLO)	Apr 2, 2024	May, 2024	~ > 53	Union Recognition, UHC Exclusion	Non-implementation of 2021 recognition agreement, exclusion from UHC staffing [52–54]
Clinical Officers (KUCO)	Apr 1, 2024	Jul 8, 2024	99	CBA implementation, UHC Contract terms, Intern management	Stalled CBA talks, prolonged short-term contracts for COs under UHC and Global Fund contracts, unfulfilled allowances, salary standardisation, and intern placements [55,56]
Clinical Officers (KUCO)	Feb 18, 2025	Mar 12, 2025	22	CBA implementation, Systemic exclusion	Non-enforcement of 2024 RTWF; failure of the enforcement of their CBA pending since 2017; exclusion of COs and their facilities from the SHA system [57–59]

Sources: Author’s compilation from sources cited.

Notes: CBA = Collective Bargaining Agreement, KMPDU = Kenya Medical Practitioners and Dentists Union, KNUMLO = Kenya National Union for Medical Laboratory Officers, KNUNM = Kenya National Union of Nurses and Midwives, KUCO = Kenya Union for Clinical Officers, PPE = Personal Protective Equipment, RTWF = Return-To-Work-Formula, SHA = Social Health Authority, UHC = Universal Health Coverage.

Strike duration and complexity also increased over time, with large-scale strikes lasting up to 150 days and involving multiple cadres, exemplified by the 100-day doctors strike in 2016–2017, the 150-day nurse strike in 2017, and the 99-day clinical officer strike in 2024 (Fig 1). These prolonged disputes may reflect persistent worker grievances alongside challenges in dispute-resolution mechanisms and agreement enforcement [28,29,44].

**Fig 1:**
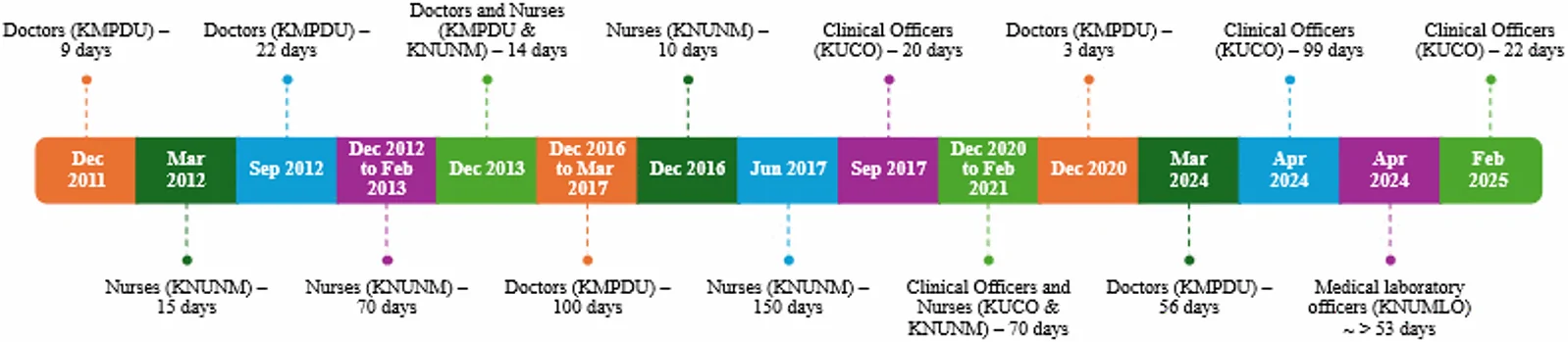
Timeline and duration of nationwide healthcare worker strikes in Kenya, 2011-2025.

The focus of strike grievances also evolved from remuneration and union-recognition concerns to broader governance concerns, including non-implementation of Collective Bargaining Agreements (CBAs) and Return-to-Work Formulas (RTWFs), workforce management, career progression, and accountability challenges [44,66–69].

Table 3 also reveals the persistence of CBA and RTWF implementation disputes across multiple cadres and strike periods. The recurrence of these disputes and repeated agreements may indicate that the challenge extends beyond dispute resolution alone to include implementation and accountability concerns. Repeated cycles of negotiation, contested implementation, and industrial action could be understood as reflecting underlying governance challenges within Kenya’s devolved health system, although alternative labour relations explanations remain important.

More recent strikes reveal the emergence of new tensions associated with health sector reforms. In 2024 and 2025, disputes increasingly centred on UHC-related contracting arrangements, intern placements, and concerns regarding exclusion of clinical officers and their facilities from the Social Health Authority (SHA) system (Table 3). These grievances highlight the labour relations challenges that can accompany major health system and service-delivery reforms.

Taken together, these trends suggest that persistent governance and institutional challenges may be an important part of understanding recurrent strikes within Kenya’s decentralised health system. However, the present study cannot determine the relative contribution of these factors compared to union mobilisation, union strategies, collective bargaining dynamics, professional interests, or other labour relations processes.

Kenya’s legal framework provides extensive protections for labour rights and multiple mechanisms for dispute resolution. However, Table 4 suggests that this may be less a consequence of the absence of legal or institutional provisions than of challenges associated with implementation, coordination, and enforcement across institutions [44,67]. At the same time, the existence of repeated industrial action despite these mechanisms may also reflect evolving union strategies, shifting bargaining dynamics, and the growing organisational capacity of HW unions.

**Table 4:** Overview of Kenyan labour laws shaping health workforce relations.

Category	Labour law	Scope and Key provisions	Relevance to Health workforce governance
Foundational Rights & Judicial Framework	Constitution of Kenya, 2010	• Guarantees fair remuneration, reasonable working conditions, freedom of association, trade union participation, and collective bargaining (Article 41). • Establishes the Employment and Labour Relations Court (ELRC) with exclusive jurisdiction over labour disputes (Article 162) [36].	Anchors both the right to strike and the right to health, creating a legal tension central to HW disputes.
	Employment and Labour Relations Court (ELRC) Act, 2011 (revised 2014)	• Establishes the ELRC as a superior court of record on employment and labour disputes. • Mandates arbitration of employment and labour disputes. Empowers the court to enforce workers’ rights, resolve industrial actions, and interpret CBAs [70,71].	Provides judicial enforcement of HW rights and dispute resolution, but courts often refer parties back to negotiation.
Employment Standards & Dispute Resolution	Employment Act, 2007 (revised 2024)	• Defines the fundamental rights of workers and sets minimum standards for working conditions (hours, leave, termination procedures). • Protects against unfair dismissal; requires written contracts and clear employment terms. • Provides a statutory dispute resolution framework: conciliation first, then referral to ELRC [72].	Governs HW contracts, dismissal protections, and dispute pathways; often invoked in individual disputes.
	Labour Relations Act, 2007 (revised 2022)	• Regulates trade unions and employers’ organisations. • Promotes freedom of association and collective bargaining. • Establishes mechanisms for orderly collective dispute resolution [73].	Governs HW union registration, CBAs, and legality of strikes; absence of a formal Conciliation and Mediation Commission weakens enforcement.
Institutional & Policy Frameworks	The Labour Institutions Act, 2007 (revised 2023)	• Consolidates labour legislation and establishes key institutions (National Labour Board, Wages Councils) • *Guides* labour *policy, sets minimum wage standards, resolves disputes* [74].	Shapes wage policy and institutional oversight affecting HW pay and conditions; overlaps with county-level mandates.
Workplace Safety & Welfare	Occupational Safety and Health Act, 2007	• Ensures safety, health, and welfare of all persons at workplaces. • Obligates employers to maintain safe environments, implement risk prevention, and provide training and protective equipment [75].	Central to HW safety disputes, especially during COVID-19 (e.g., PPE shortages, risk allowances).

Sources: Author’s compilation from sources cited.

The analysis also highlights a persistent tension between the constitutional right to strike and the right to health. Although health is designated as an “essential service,” the legal framework provides limited operational guidance on maintaining continuity of care during industrial action [27,67,73], which may contribute to disputes that progress from negotiation to litigation and emergency interventions [29,44,76].

Devolution has further complicated these dynamics by creating overlapping responsibilities and accountability challenges across levels of government [8,40,42]. Consequently, negotiated agreements are often inconsistently implemented, contributing to recurring labour disputes despite the existence of formal dispute-resolution mechanisms [29,44,66,68].

### Dispute resolution gaps and enforcement weaknesses

Our analysis suggests that Kenya’s legal framework provides multiple mechanisms for managing labour disputes, including conciliation, mediation, arbitration, and judicial review. However, their implementation appears uneven across the health sector [44,67,71,73]. As shown in Table 4, constitutional guarantees, labour statutes, and institutional mandates coexist, yet there remains limited operational framework integration of these mechanisms for managing labour disputes within a devolved health system context.

In practice, disputes often bypass early-stage conciliation and progress to litigation or prolonged industrial action [29,67]. Our findings suggest that overlapping responsibilities between national and county actors, coupled with challenges in enforcing negotiated outcomes, may limit the effectiveness of existing dispute-resolution mechanisms. Judicial interventions often focus on procedural compliance but may not fully resolve underlying grievances, while implementation of court decisions appears inconsistent in some instances [28,76,77]. One possible consequence is that dispute management has become largely reactive, which may contribute to strained relationships between health workers and government actors.

Our analysis suggests that the absence of a permanent and institutionalised conciliation and mediation body may represent an important gap within Kenya’s labour dispute resolution architecture [67,76,78]. Although the Labour Relations Act provides for conciliation, reliance on ad hoc appointments by the Ministry of Labour may contribute to delays, limited continuity, and perceptions of politicisation [44,67,71,73]. These shortcomings may undermine confidence in mediation processes and reduce incentives for early engagement by disputing parties [27,28,49].

As a result, many labour disputes often remain unresolved until they culminate in strikes or litigation, at which point resolution becomes more politically contentious and socially costly. The absence of institutional memory, technical capacity, and enforcement authority may make it more difficult to develop predictable and trusted pathways for dispute resolution, further entrenching adversarial labour relations within the health sector [28,29,44].

### Impact on service delivery and systemic challenges

Our study found that HW strikes have consistently disrupted service delivery across Kenya’s public health system, particularly in the absence of effective contingency mechanisms [7,9,10,79]. Across multiple strike periods, documented impacts included reductions in outpatient attendance, maternal and child health services, emergency care, and chronic disease management [7,10,80]. These effects were most pronounced in rural and low-income settings, where access to alternative sources of care is limited *[*9,10,67*]*.

Rather than isolated events, these service disruptions expose underlying weaknesses in health system resilience and planning. Limited implementation of minimum service arrangements, coupled with ineffective dispute-resolution mechanisms and reactive governance responses, may have increased the severity and duration of service disruptions associated with industrial action on vulnerable populations *[*10,27,29,81*]*. Over time, recurrent service interruptions have eroded public trust in public health services and created additional challenges for efforts to advance universal health coverage *[*29,44,82,83*].*

### Cross-cutting patterns and governance dynamics

Across the findings, we identified several recurring patterns that may help explain the persistence of recurrent labour unrest within Kenya’s health sector. Fragmented authority arrangements can create ambiguity over responsibility for workforce management and implementation of negotiated agreements across national and county institutions. Such ambiguity may weaken accountability and complicate efforts to address grievances in a timely and coordinated manner.

Our findings further suggest that where implementation challenges persist, confidence in formal dispute-resolution mechanisms may be reduced. In such circumstances, industrial action may increasingly be viewed by HW organisations as a viable strategy for advancing claims or securing government attention.

Our findings also suggest that repeated cycles of negotiation, industrial action, and contested implementation may contribute to ongoing mistrust between negotiating parties. Rather than representing a simple causal chain, these dynamics may interact over time in ways that reinforce labour tensions and compliance in future negotiations. From this perspective, recurrent strikes may be understood as emerging from the interaction of governance arrangements, labour relations processes, organisational interests, and political context rather than any single factor alone.

## Discussion

This analysis suggests that recurrent health worker strikes in Kenya may be understood not only as episodic labour disputes but also as reflecting broader governance and institutional challenges within a devolved health system. A central finding is the persistence of recurrent labour unrest despite repeated reforms, negotiated agreements, and dispute-resolution efforts. Recurrent disputes over CBA and RTWF implementation may suggest that challenges in translating negotiated commitments into sustained action are one factor that may contribute to strike recurrence, alongside broader labour relations and collective bargaining dynamics. Collectively, the findings highlight interconnected governance, accountability and implementation challenges that appear relevant to understanding the recurrence of labour disputes despite extensive legal and policy reforms, with important implications for health system resilience, accountability, and equity in Kenya and other decentralised health systems, particularly in LMIC settings.

### The constitutional dual-rights paradox and its governance implications

Kenya’s simultaneous constitutional protection of the right to strike and the right to health creates a structural governance paradox that appears to remain operationally unresolved in practice [4,17,23,25]. While Articles 41 and 43 of the 2010 Constitution guarantee both labour rights and access to healthcare, the findings suggest that there are limited institutional mechanisms for reconciling these rights in essential services, creating challenges for their operationalisation during periods of industrial action [36,67].

In practice, unions, government actors, and courts often appear to negotiate this tension reactively, during periods of crisis, rather than through clearly defined governance arrangements. This reflects challenges that can arise across health systems but may be particularly pronounced in LMIC contexts where institutional and implementation capacity is often constrained [1,33].

Judicial interventions have attempted to balance these competing rights, at times permitting industrial action while mandating minimum service provision. However, enforcement remains inconsistent, and reliance on litigation may be limited in its ability to sustain service continuity or build trust between stakeholders [44,84]. These dynamics may contribute to an operationally fragile environment in which maintaining service continuity can be challenging, with potential implications for health system resilience and equitable access to care for populations that rely heavily on public healthcare services [7,20,44].

Addressing this paradox may require moving beyond constitutional guarantees towards policy instruments that more explicitly balance labour rights with continuity of care, including enforceable minimum service protocols and structured social dialogue processes [5,25].

### Institutional challenges in labour dispute management

While labour disputes are inherently shaped by bargaining processes, union strategies, and professional interests, our findings suggest that institutional and governance challenges may also contribute to strike recurrence by complicating dispute resolution and agreement implementation [9,27,28].

Although Kenya’s legal framework provides multiple avenues for dispute resolution, including conciliation, mediation, arbitration, and judicial review, the recurrent nature of disputes raises questions about how effectively these mechanisms operate in practice. Fragmentation, uneven implementation, and enforcement challenges may reduce confidence in formal dispute-resolution processes and contribute to repeated industrial action [29,67,71]. The absence of a dedicated, independent conciliation and mediation institution has contributed to delays, ad hoc appointments, and politicisation, particularly in the health sector [76,78]. As a result, disputes frequently escalate directly to litigation or prolonged industrial action before meaningful resolution occurs [28,77]. Courts often refer parties back to negotiation, but without adequate mechanisms to monitor or enforce settlements, cycles of non-compliance and mistrust persist [29,67].

This interpretation is consistent with evidence from other sub-Saharan Africa contexts suggesting that institutionalised mediation systems contribute to more effective labour dispute management by reducing strike frequency and duration by strengthening confidence in negotiated settlements [84–88]. Kenya’s continued reliance on reactive legal processes may reflect a governance gap that could undermine accountability and predictability in labour relations.

### Devolution without corresponding investment in HRH management capacity increases the risk of recurrent labour unrest

The transfer of health workforce management responsibilities to county governments without corresponding investment in HRH management capacity may have created conditions that contributed to recurrent labour disputes [8,40,41]. While devolution was intended to improve responsiveness and equity, it also fragmented authority over recruitment, remuneration, promotion, and discipline across 47 counties with widely varying technical and fiscal capacities [42,43]. Many counties struggle with delayed salaries, stalled promotions, inconsistent CBA implementation, and weak workforce planning systems [41,44].

These challenges are consistent with what has been described in the literature as principal-agent problems in decentralised systems. Where subnational actors operate with divergent incentives and limited oversight [33]. Similar patterns have been observed in other decentralised health systems, particularly in LMIC settings where institutional strengthening has not kept pace with governance reforms [1,12].

Consistent with findings by Waithaka et al. [9] and Koon [43], our analysis suggests that devolution redistributed authority for workforce management without fully clarifying accountability for implementation. Similarly, Irimu et al. [10] and Scanlon et al. [12] demonstrate that recurrent industrial action often occurs within broader contexts of governance and coordination challenges that interact with concerns regarding remuneration, working conditions, and collective bargaining.

Collectively, these studies suggest that recurrent strikes persist when responsibilities for negotiating, financing, implementing, and enforcing agreements are distributed across actors with differing mandates, incentives, and capacities. These governance arrangements may have contributed to uneven employment conditions, persistent implementation gaps, and recurring labour disputes [9,10,41]. Strengthening workforce management capacity and intergovernmental coordination will be essential if devolution is to improve responsiveness without undermining workforce stability.

### Recurrent non-implementation of negotiated agreements may be indicative of underlying accountability challenges

One of the most striking findings of this study is the persistence of disputes over CBA and RTWF implementation across multiple cadres, strike episodes, and political administrations [9,28,29,44]. Their recurrence over more than a decade suggests that the challenge extends beyond negotiation of settlements to their implementation and enforcement.

Agreements are routinely reached during periods of crisis, yet many commitments remain partially fulfilled or unfulfilled, allowing the same grievances to resurface in subsequent disputes [7,9,10,29]. This pattern may be indicative of broader governance and accountability challenges within Kenya’s devolved health system. Responsibility for negotiating, financing, implementing, and enforcing workforce agreements is distributed across multiple actors, including national ministries, county governments, the Treasury, and the SRC [10,28,29,67,69]. While national actors frequently participate in negotiations, implementation responsibilities often rest with county governments operating under varying fiscal, administrative, and political constraints [10,29].

These arrangements may create accountability gaps and principal-agent challenges that can complicate implementation and diffuse responsibility for delivery. Agreements are often negotiated to end industrial action, restore services, and relieve immediate political and public pressure [10,29], while the fiscal and administrative costs of implementation are deferred, contested, or shifted across institutions [10,29]. These arrangements may create incentives for short-term settlement of disputes without corresponding incentives for long-term sustainability, allowing negotiated agreements to function as crisis-management tools rather than durable solutions.

Collectively, the findings suggest that recurrent strikes may involve more than failures in negotiation processes alone. They reveal a system in which commitments are repeatedly made without corresponding mechanisms to ensure implementation, monitoring, or enforcement. The findings suggest that the persistence of labour unrest may be associated not only with the content of negotiated agreements, but also with challenges in their implementation and follow-through.

From a complex adaptive systems perspective, recurrent strikes may be understood as an emergent property of interactions among multiple actors operating within a fragmented governance environment. Efforts to resolve immediate disputes often fail to alter the underlying incentives and accountability arrangements that generate conflict, potentially contributing to recurring cycles of negotiation, partial implementation, and industrial action.

### The absence of minimum service agreements may represent an important resilience and continuity-of-care challenge

Our findings suggest that the absence of enforceable minimum service agreements may pose important challenges for health system resilience and the protection of equitable access to care during periods of industrial action [27,67].

In the absence of structured contingency planning, strikes have repeatedly disrupted service delivery, including emergency, maternal, and chronic care services [7,15,79]. These impacts are likely to be felt most acutely by poor and rural populations who have limited access to alternative sources of care [13,14].

Evidence from other settings suggests that minimum service agreements can help reconcile labour rights with service continuity by safeguarding critical essential functions during industrial action [5,6]. Kenya’s reliance on ad hoc and inconsistently applied arrangements may be consistent with a broader pattern in which legal and policy commitments are not translated into effective implementation, reinforcing the governance and accountability gaps that characterise many recurrent labour disputes.

Embedding minimum service requirements within labour and health policy frameworks could help operationalise the balance between labour rights and service continuity while potentially strengthening protection for vulnerable populations during industrial action.

A consistent pattern emerging from these findings is that responsibilities for negotiating, financing, implementing, and enforcing workforce agreements are dispersed across institutions with differing mandates and incentives. Such arrangements may contribute to the persistence and recurrence of labour disputes. Consequently, some reforms may focus on resolving immediate disputes without fully addressing the broader governance and implementation challenges that can contribute to future tensions.

## Implications for health system reform and resilience

Taken together, our findings suggest that recurrent HW strikes in Kenya may reflect an interaction between labour grievances, collective bargaining processes, and persistent governance and implementation challenges. The findings suggest that legal protections and negotiated agreements may be insufficient in the absence of clear accountability arrangements, coordinated mandates, and effective implementation mechanisms [8,33].

Priority reforms include strengthening accountability for negotiated commitments, establishing independent dispute resolution institutions, enhancing intergovernmental coordination, harmonising HRH management systems across levels of government, strengthening workforce management capacity, and institutionalising minimum service protections. Without such reforms, conditions associated with recurrent labour unrest may remain a persistent feature of Kenya’s devolved health system, with consequences for service delivery, equity, and progress toward UHC [11,21].

Anchoring future reforms in accountability, equity, and system resilience may help strengthen labour relations and improve health service delivery [12,14].

## Study limitations and Future research

This study has several limitations that should be considered when interpreting its findings. First, it relies exclusively on secondary data sources, including policy documents, legal documents, media reports, and union communications. While these enabled broad coverage of developments across Kenya’s devolved health system, they limited insight into lived experiences, informal negotiation processes, and frontline perspectives that shape labour disputes. Second, the use of publicly available sources introduces potential reporting and framing biases. Government documents may understate governance challenges, while media and union sources may emphasise conflict narratives. Although triangulation across multiple data types helped mitigate this, some interpretive bias may remain. Third, the study’s temporal focus on the 2010–2025 post-devolution period strengthens relevance to current governance dynamics but may not fully capture longer-term historical trends or emerging reforms, limiting generalisability beyond this context. Fourth, comparative insights from other LMICs are illustrative rather than based on systematic cross-country analysis and should therefore be interpreted cautiously.

Future research should incorporate primary qualitative data from health workers, union leaders, and policymakers to better understand negotiation dynamics and informal governance practices. Mixed-methods studies linking labour unrest to service delivery outcomes, as well as comparative analyses across decentralised health systems, would further strengthen the evidence base and inform context-specific policy solutions.

## Conclusion

This study suggests that recurrent health worker strikes in Kenya cannot be understood solely as isolated labour disputes and may also reflect broader systemic challenges in health workforce governance, accountability, and implementation. Beyond reflecting episodic labour disputes, the persistence of strikes despite repeated reforms, negotiated agreements, and dispute-resolution efforts may point to broader governance, accountability, and institutional coordination challenges. The findings highlight three interrelated challenges that may contribute to these disruptions: unresolved tensions between the constitutional rights to strike and to health, fragmented and weakly enforced dispute resolution mechanisms, and uneven human resource management capacity under devolution.

The findings suggest that greater emphasis on proactive institutional reform, alongside crisis management measures, may help address these challenges. Establishing an independent and well-resourced dispute resolution mechanism may strengthen the credibility and timeliness of labour negotiations. Clearer delineation of roles across national and county governments, alongside harmonised HRH management systems, is essential to reduce fragmentation and strengthen accountability. Greater emphasis on implementing and enforcing negotiated agreements, including CBAs and RTWFs, may be important for reducing recurrent cycles of labour unrest. Enforceable minimum service agreements may also help safeguard essential services while respecting labour rights.

Kenya’s experience highlights broader lessons for decentralised health systems and may be particularly relevant to LMICs pursuing governance reforms. The findings suggest that legal protections alone may be insufficient and that resilient health systems may benefit from coherent institutional arrangements, aligned incentives, effective implementation processes, and sustained investment in workforce management to ensure equitable and uninterrupted access to care. Fundamentally, sustainable solutions may depend not only on reaching agreements, but also on strengthening the institutional capacity, accountability, and implementation mechanisms needed to translate commitments into sustained action.
